# Aged Brains Express Less Melanocortin Receptors, Which Correlates with Age-Related Decline of Cognitive Functions

**DOI:** 10.3390/molecules26206266

**Published:** 2021-10-16

**Authors:** Yang Zhou, Monica K. Chawla, Jose L. Rios-Monterrosa, Lingzhi Wang, Marc A. Zempare, Victor J. Hruby, Carol A. Barnes, Minying Cai

**Affiliations:** 1 Department of Chemistry & Biochemistry, The University of Arizona, Tucson, AZ 85721, USA; yangzhou@email.arizona.edu (Y.Z.); riosmonterrosa@email.arizona.edu (J.L.R.-M.); lzwang@email.arizona.edu (L.W.); hruby@email.arizona.edu (V.J.H.); 2 Evelyn F. McKnight Brain Institute, The University of Arizona, Tucson, AZ 85721, USA; MChawla@nsma.arizona.edu (M.K.C.); mzempare@email.arizona.edu (M.A.Z.); carol@nsma.arizona.edu (C.A.B.); 3 Division of Neural Systems, Memory & Aging, The University of Arizona, Tucson, AZ 85721, USA; 4 Department of Psychology, Neurology and Neuroscience, The University of Arizona, Tucson, AZ 85721, USA

**Keywords:** aging, melanocortin receptor, MC1R, MC3R, MC4R, MC5R, cognitive decline, hippocampus, frontal cortex

## Abstract

Brain G-protein coupled receptors have been hypothesized to be potential targets for maintaining or restoring cognitive function in normal aged individuals or in patients with neurodegenerative disease. A number of recent reports suggest that activation of melanocortin receptors (MCRs) in the brain can significantly improve cognitive functions of normal rodents and of different rodent models of the Alzheimer’s disease. However, the potential impact of normative aging on the expression of MCRs and their potential roles for modulating cognitive function remains to be elucidated. In the present study, we first investigated the expression of these receptors in six different brain regions of young (6 months) and aged (23 months) rats following assessment of their cognitive status. Correlation analysis was further performed to reveal potential contributions of MCR subtypes to spatial learning and memory. Our results revealed statistically significant correlations between the expression of several MCR subtypes in the frontal cortex/hypothalamus and the hippocampus regions and the rats’ performance in spatial learning and memory only in the aged rats. These findings support the hypothesis that aging has a direct impact on the expression and function of MCRs, establishing MCRs as potential drug targets to alleviate aging-induced decline of cognitive function.

## 1. Introduction

Dysfunction of a number of brain G-protein coupled receptor (GPCR) systems has been demonstrated to be associated with age-related cognitive decline, which could be followed by progressive cognitive impairments and eventually lead to neurodegenerative diseases such as Alzheimer’s disease (AD) [1]. The M_1_ muscarinic acetylcholine receptor, which exerts neuroprotective effects upon activation [2,3], was demonstrated to have significantly reduced G-protein-coupling in aged, memory-impaired rats. This decline was significantly correlated with the severity of their memory impairments [4]. Further dysfunction in M_2_ and M_4_ receptors was found in individuals with the amnestic mild cognitive impairment (aMCI), an observation that persists into AD [5,6,7,8]. Similarly, a significant decrease in the expression of the GABA_B_ receptor in the prefrontal cortex was found in aged rats, and was found to be negatively associated with working memory performance in the older animals [9,10]. The 5-HT_6_ receptor and the mGlu2/3 receptors also showed age-dependent expression levels in the central nervous system [11,12]. Thus, GPCRs have become important drug targets to be explored for restoring cognitive function in aged people as well as in MCI and Alzheimer’s disease patients [13,14,15,16].

The melanocortin receptor (MCR) system consists of five distinct receptors (MC1R-MC5R) that regulate many physiological functions, including skin pigmentation, modulation of immune responses, sexual function, glucose metabolism, energy homeostasis, feeding behavior and exocrine secretion [17]. The MC3R and the MC4R are mainly expressed in a number of brain regions [18,19,20,21]. The MC1R and the MC5R are highly expressed in skin melanocytes and exocrine glands, respectively, while their expression is also found in the brains of humans and mice, as shown by the Human Protein Atlas [22] and the Allen Mouse/Human Brain Atlas [23,24]. MCRs in the brain have long been associated with cognitive function, following observations that treatments with the endogenous MCR agonists melanocyte-stimulating hormones (MSHs) can improve learning, memory and attention in human and rodents [25,26,27]. Activation of the MC4R has been demonstrated to induce cognitive recovery and alleviate synaptic plasticity in several AD mouse models [28,29,30,31]. The exact relationship between different MCR subtypes and memory loss as a result of aging, however, has not been well studied. Moreover, even though all MCRs share the same downstream signaling of Gs-cAMP-PKA, potential contributions of the MC1R, MC3R and MC5R to cognitive function has not been examined separately.

To determine how aging affects MCR expression, we performed total and subtype specific radioligand binding assays on six different brain regions of young (6 months) and aged (23 months) rats. The spatial learning and memory of these rats was assessed through the spatial version of the Morris water maze before the rats were sacrificed, and the correlation between MCR expression levels in different brain regions and the rats’ cognitive performance was analyzed to shed light on the MCR subtypes that may contribute to enhanced cognitive function.

## 2. Results

### 2.1. Radioligand Binding Assays to Determine Brain Region-Specific MCR Expression in Young (6 Mon) and Aged (23 Mon) Rats

**Figure 1:**
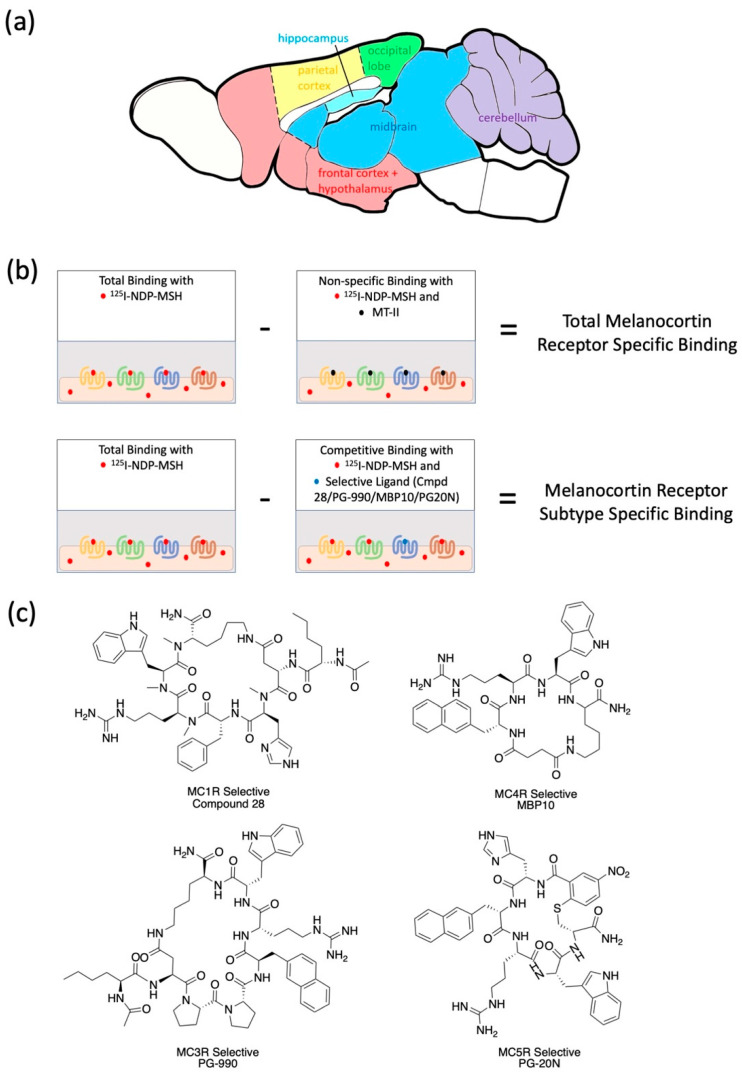
The methods and data processing of [^125^I]-NDP-MSH radioligand binding assay to determine the total and subtype specific melanocortin receptor binding in different rat brain regions. (**a**) A sagittal view of a rat brain showing the six different regions of frontal cortex + hypothalamus, parietal cortex, occipital cortex, hippocampus, midbrain and cerebellum that were studied in this study. (**b**) Schematic of the [^125^I]-NDP-MSH radioligand binding assays to determine the total and subtype specific melanocortin receptor binding in different brain regions of rats. (**c**) Melanocortin receptor subtype selective ligands (Compound 28, MC1R selective ligand [33]; PG990, MC3R selective ligand [34]; MBP10, MC4R selective ligand [35]; PG20N, MC5R selective ligand [36] used to determine subtype specific melanocortin receptor binding).

To test how aging affects the expression of MCRs in different brain regions, two groups of male F344 rats of different ages (6 months or 23 months) were tested on the Morris water maze to determine spatial learning performance, and were sacrificed one week after behavior testing. The rats’ brains were dissected into six different brain regions of the frontal cortex/hypothalamus, the parietal cortex, the occipital lobe, the hippocampus, the midbrain and the cerebellum (Figure 1a). The brain samples were homogenized, and membrane proteins were extracted. To determine the total amount of MCRs in different brain regions of the young and aged rats, radioligand binding assays with [^125^I]-NDP-α-MSH were performed (Figure 1b). Membrane protein extracts from different brain regions were incubated with [^125^I]-NDP-α-MSH. Unbound radioligands were washed away and the amount of bound [^125^I] was measured as total binding, which includes binding of [^125^I]-NDP-α-MSH to MCRs (specific binding) and binding of [^125^I]-NDP-α-MSH to other membrane protein extracts (non-specific binding). The amount of non-specific binding was further measured by adding excess amount of melanotan-II (MT-II), which is known to compete for the ligand binding sites of MCRs and completely knock off specific [^125^I]-NDP-α-MSH binding at high concentration [32]. Radiation counts from non-specific binding were subtracted from the total binding counts, yielding radiation counts that come from specific binding (Figure 1b).

**Figure 2:**
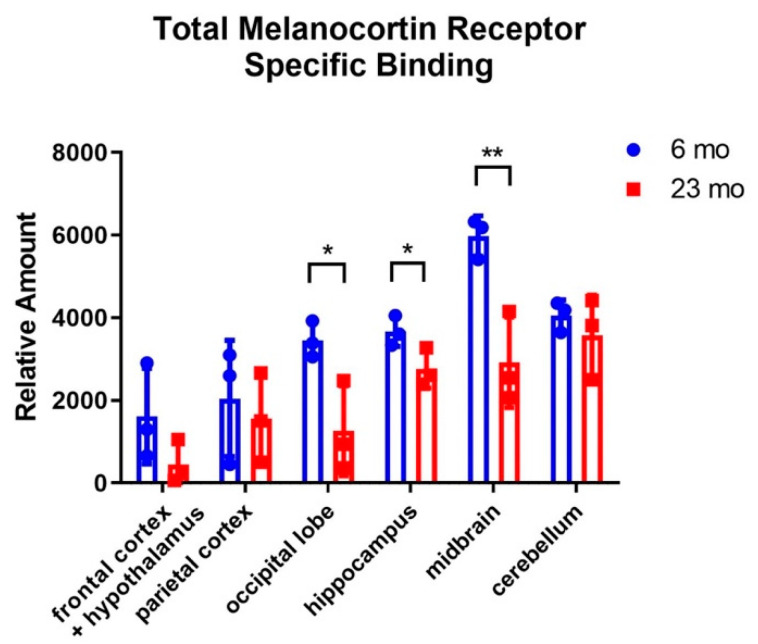
Total melanocortin receptor specific binding of [^125^I]-NDP-MSH to different brain regions of 6 mo (*n* = 6) and 23 mo (*n* = 6) rats. Statistical significance was calculated by a Student’s*t*-test. * indicates *p* < 0.05. ** indicates *p* < 0.01.

The total MCR specific binding results suggest that MCRs are expressed in many different brain regions of the rat, with relatively high levels of expression in the hippocampus, the midbrain and the cerebellum (Figure 2).

Comparing between young (6 mo) and aged (23 mo) rats, the aged rats have significantly lower levels of MCR expression in the occipital lobe, the hippocampus and the midbrain.

To identify the MCR subtypes that have differential expression levels in young (6 mo) and aged (23 mo) rats, radioligand binding assays were performed with four MCR selective ligands to selectively bind to and competitively inhibit the binding of [^125^I]-NDP-α-MSH to the MC1R, the MC3R, the MC4R or the MC5R (Figure 1b,c). Radiation counts from competitive binding with subtype selective ligands were subtracted from the total binding counts, yielding radiation counts that come from subtype specific binding (Figure 1b).

**Figure 3:**
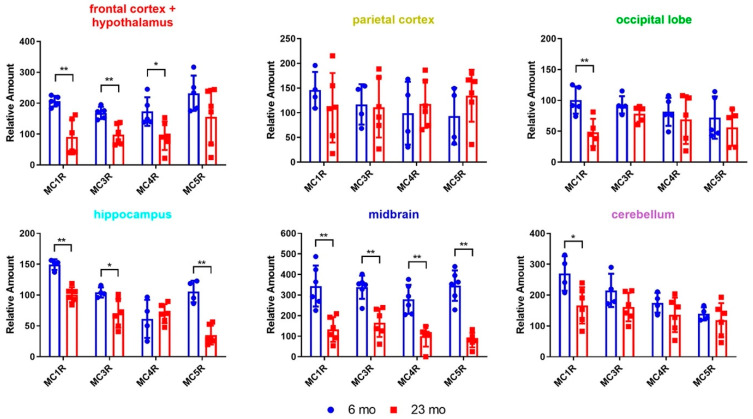
Subtype specific binding of ^125^I-NDP-MSH to different brain regions of 6 mo (*n* = 6) and 23 mo (*n* = 6) rats. Statistical significance was calculated by a Student’s *t*-test. * indicates *p* < 0.05. ** indicates *p* < 0.01.

The results suggest that all MCR subtypes are expressed in all the regions of the brain studied here (Figure 3). The MC1R has significantly lower expression in all the brain regions except for the parietal cortex. Aged rats showed significantly reduced MC3R expression in the frontal cortex/hypothalamus, the hippocampus and the midbrain regions compared to the young rats. MC4R expression is also reduced in aged rats compared to young rats in the frontal cortex/hypothalamus and the midbrain. Surprisingly, the MC5R has 3-4-fold higher expression in the hippocampus and the midbrain region of young rats comparing to the aged rats. These results demonstrate that aging has a direct impact on the expression of MCRs in different brain regions, which may further influence the cognitive functions that are regulated by the MCRs.

### 2.2. Correlations between Rats’ Performance in Spatial Learning and Memory and the Expression of MCRs in Their Brain

**Figure 4:**
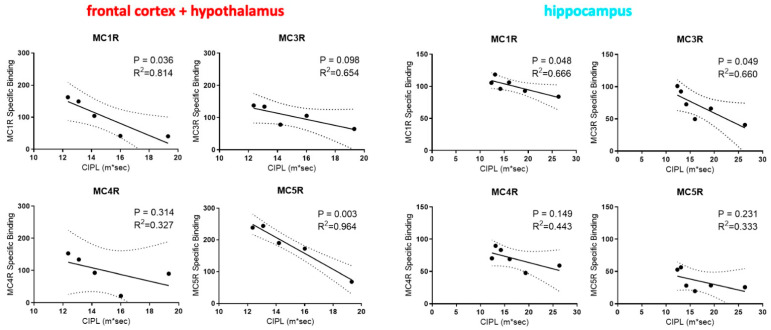
Linear correlations between the expression levels of different melanocortin receptor subtypes in the hippocampus and the frontal cortex/hypothalamus regions in the brains of aged (23 mo) rats and their performance in spatial learning and memory as measured by the Correlated Integrated Path Length (CIPL) in the Morris swim task.

**Figure 5:**
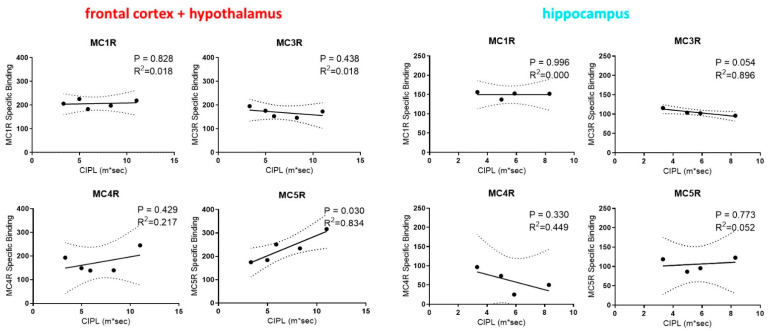
Linear correlations between the expression levels of different melanocortin receptor subtypes in the hippocampus and the frontal cortex/hypothalamus regions in the brains of young (6 mo) rats and their performance in spatial learning and memory as measured by the Correlated Integrated Path Length (CIPL) in the Morris swim task.

To assess whether the expression levels of different MCR subtypes are associated with differences in the cognitive functions, we performed regression analysis between the levels of different MCR subtypes and the spatial learning performance of rats as indicated by their Correlated Integrated Path Length (CIPL) measure on the Morris water maze. We focused only on the frontal cortex/hypothalamus and the hippocampus regions for the correlation analysis due to their importance in cognitive functions and in this task [37]. Interestingly, MC1R and MC3R expression in the hippocampus along with the MC1R and the MC5R expression in the frontal cortex/hypothalamus are all significantly correlated with the CIPL within aged rats (Figure 4). In contrast, no statistically significant correlations were found between the MCR expression levels in the frontal cortex/hypothalamus and the hippocampus regions in young rats and their behavior performance (Figure 5). The expression of the MC5R in the frontal cortex, in particular, showed strong correlation with the CIPL of aged rats, with a *p*-value of 0.03. The expression of MC4R in both brain regions did not show correlations with the behavior performance. These results suggest that MCR signaling and function may differ between young and aged rats, and that higher expression levels and stronger signaling of MCRs in the frontal cortex and the hippocampus may lead to improvement in cognitive functions in aged rats but not in young rats.

## 3. Discussion

Aging-related cognitive decline is an important biological process that remains to be fully understood. If left untreated, mild cognitive impairments can progress into Alzheimer’s disease or other types of dementia. Many brain GPCRs have been shown to play important roles in aging-related cognitive decline, and thus serve as drug targets for potential treatments. Following reports that activation of the MCRs leads to improvement in cognitive function in normal and AD disease model rodents, this research aims to understand the role of MCRs in cognitive functions during the normal aging process. We made the interesting observation that in aged rats, the expression levels of several MCR subtypes in the frontal cortex and the hippocampus have statistically significant correlations with performance in spatial learning and memory, whereas no correlations were observed in young rats. Similar observations were made on the muscarinic acetylcholine receptor and the GABA_B_ receptor that correlations between the expression levels of these GPCRs and performance in cognitive functions can be observed in aged rodents but not young ones [4,9]. One explanation is that the GPCRs in young brains work redundantly so that not a single GPCR effectively predicts cognitive performance. During aging the expression of brain GPCRs deviates from the normal range, and may in part be responsible for the decline in cognitive function observed. Together, these observations suggest that the signaling and biology of GPCRs are different in young and aged brains, further stressing the importance to study the biology of aged brains.

Traditionally, the MC3R and the MC4R are known to regulate food intake and energy homeostasis in the hypothalamus, whereas the MC1R and the MC5R are mainly considered to exert their functions outside the brain [17]. Using radioligand binding studies, we identified the expression of different MCR subtypes in all the brain regions examined. Our results are consistent with the mRNA expression data from the Human Protein Atlas and the Allen Brain Atlas that all MCR subtypes are expressed in the brain, with higher levels of expressions especially in the cerebellum, the hippocampus and the basal ganglia (included in the midbrain region in this study) [22,23,24]. Demonstrating the presence of these receptors in the brain, our work lays the foundation for understanding their specific functions in the future.

The protein levels in the brain can be directly measured by radioligand binding experiments, or by antibody-antigen measurements such as Western blot or enzyme-linked immunosorbent assay (ELISA). The radioligand binding assays are widely used to quantify GPCRs in the brain when highly selective ligands are available [6,7]. Recent advances in the developments of subtype selective ligands to all MCR subtypes (reviewed in [17,38]) enabled us to perform radioligand binding assays to quantify the expression levels of MCRs for the first time. The radioligand binding assays also allow us to directly compare the expression levels among MCR subtypes, which could be very difficult if using an antibody-based technique due to different antibodies having different affinities to the target protein and to their secondary antibodies. Alternatively, the protein expression levels can be estimated by the mRNA levels using techniques such as in situ hybridization and RNA-seq. However, the mRNA levels are not sufficient to predict protein levels since they do not capture any post-transcriptional processes such as protein translation, post-translational modifications or protein degradation [39].

Within the hippocampus region, the MC4R has been the focus of study, especially in the context of AD. The MC4R was shown to regulate hippocampal synaptic plasticity in healthy mice as well as in an AD mouse model, through the cAMP-PKA signaling pathway [28,40]. Further studies demonstrated that MC4R activation protects against the disease progression in different AD mouse models [29,30,31,41]. In this study, we demonstrated that the MC4R might play a less important role in the context of normative aging. The MC4R expression level is not significantly different between young and aged rats, and no correlation was observed between the MC4R expression level and the performance of rats in spatial learning and memory. Instead, the MC1R and the MC3R, which have higher expression levels in the hippocampus than the MC4R and showed statistically significant correlations with the performance of aged rats in spatial learning and memory, may be better drug targets for the aging-related cognitive decline. Correlations between MC1R expression levels and cognitive decline during aging might be due to MC1R’s expression in glial cells and its ability to attenuate neuroinflammation through the Gs-cAMP-PKA pathway [42,43], as chronic inflammation has been proposed to be an important mechanism underlying cognitive decline and dementia [44]. Through antibody staining provided by the Human Protein Atlas, the MC3R was shown to be expressed exclusively in neuronal cells but not in glial cells within the hippocampus [22]. The MC3R may share similar physiological functions with the MC4R through the Gs-cAMP-PKA pathway if further evidence could support that the MC3R and the MC4R are expressed in the same neuron population.

## 4. Materials and Methods

### 4.1. Animals and Morris Water Maze Testing

Twelve young (6 mo) and thirteen aged (23 mo) male Fischer 344 rats were used in this study. Rats were housed individually with unrestricted access to food and water. Behavioral testing was done during the dark phase of the rat’s 12 h light and dark cycle following several days of handling.

The Morris water maze was used to examine spatial learning and memory following previously described procedures [45]. Briefly, rats were given health checks and handled in the week before training. Rats were initially placed onto the hidden platform of the water tank for 30 s, before being placed into the pool at pseudo-randomly assigned start locations at the edge of the pool. A total of six spatial trials were given per day for 4 days (24 trials total). Following the spatial trials, 6 trials were given on each of 2 days (12 trials total) in which the submerged platform was above the water’s surface (“cued trials”) to ensure that the animals did not have visual or motor defects that could influence performance. Since the start locations and swim velocity vary between animals, a corrected integrated path length (CIPL) was calculated to ensure comparability of the rats’ performance across release locations [46]. The corrected integrated path length (CIPL) values of the last 12 spatial trials were used for each age group.

### 4.2. Brain Extraction Procedures

Prior to decapitation with a guillotine, rats were anesthetized with isoflurane. Brains were quickly extracted, rinsed in ice-cold saline and dissected on a metal plate as follows. A midline cut separated the right and left hemisphere, each hemisected brain was separated into frontal cortex + hypothalamus, parietal cortex, occipital lobe, hippocampus, midbrain and cerebellum. The dissected subregions were placed in separate 2 mL microfuge tubes containing 1 mL of ice-cold 50 mM HEPES buffer plus Mammalian Protease Inhibitor Cocktail (Sigma) and quickly frozen in liquid nitrogen.

### 4.3. Membrane Preparation and Protein Quantification

Brain tissue from six major regions was homogenized for 60 s with a sawtooth homogenizer in 3 mL of membrane buffer (50 mM HEPES pH 7.5). A total of 0.1% of a mammalian protease cocktail inhibitor (Sigma) was added to the buffer immediately before beginning the extraction. Samples were then centrifuged at 4 °C for 10 min (1500 rpm) with a Beckman Ultracentrifuge. The supernatant was isolated and centrifuged again at 4 °C for 2 h (32,750 rpm) in a Beckman ultracentrifuge. After centrifugation, the pellet was re-suspended in 1.2 mL of membrane buffer and aliquots of 300 μL were stored at −80 °C. Additionally, 50uL were set aside for protein concentration determination. The protein concentration of the membrane was determined using a BCA assay (Pierce). The purple reaction product was monitored at 560 nm using an enzyme-linked immunosorbent assay plate reader (μQuant, Bio-Tek Instruments, Inc.).

### 4.4. Membrane Binding Assays

The brain samples were thawed on ice and diluted with HEPES buffer (50 mM, with 0.1% peptidase inhibitor) to a final protein concentration of 50 μg/mL. [^125^I]-NDP-α-MSH (PerkinElmer) was diluted with HEPES buffer supplemented with BSA (50 mM, with 0.1% peptidase inhibitor and 6% BSA) to a final concentration of 0.8 nM. The MC1R selective agonist compound 28 [33], MC3R selective agonist PG-990 [34], MC4R selective antagonist MBP10 [35] and MC5R selective antagonist PG20N [36] were synthesized through solid-phase peptide synthesis with over 95% purity. These selective peptides were independently diluted to 1 μM with HEPES buffer supplemented with BSA. The non-selective melanocortin receptor agonist MT-II, which was synthesized with over 95% purity, was diluted to 4 μM with HEPES buffer supplemented with BSA. A total of 25 μL of the [^125^I]-NDP-α-MSH solution was premixed with 25 μL of solution with melanocortin receptor selective ligand, MT-II or HEPES buffer in 96-well plates. A total of 50 μL of the brain sample was added to each well. Each combination of brain sample and selective/nonselective melanocortin receptor ligand was repeated two times in triplicates. The 96-well plates were incubated at 4 °C for 1 h and centrifuged at 4000 rpm at 4 °C for 10 min. Supernatants were removed. The pellets were washed with 125 μL of ice-cold HEPES buffer and centrifuged again at 4000 rpm at 4 °C for 10 min. Supernatants were removed, and 100 μL of scintillation fluid was added to each well. The [^125^I] radiation on 96-well plates were counted by Microbeta 2 (PerkinElmer). To calculate the selective [^125^I]-NDP-α-MSH binding to all melanocortin receptors, the non-selective count (with MT-II added) was subtracted from the total count (no MT-II or selective ligands). To calculate the selective [^125^I]-NDP-α-MSH binding to a certain melanocortin receptor subtype, the count with selective ligand to the melanocortin receptor subtype added was subtracted from the total count.

### 4.5. Statistical Analysis

For membrane binding assays, a Student’s*t*-test was performed to calculate the statistical differences. For comparison of behavior and biochemical assays, simple linear regression was used to assess differences between treatment groups over training trials with alpha levels set at 0.05. The data analysis and display were performed with GraphPad Prism 6.

## Data Availability

The datasets used during the current study are available from the corresponding author upon reasonable request.

## Sample Availability

Samples of the compounds are not available from the authors.
